# Assessing genetic polymorphisms using DNA extracted from cells present in saliva samples

**DOI:** 10.1186/1471-2288-11-170

**Published:** 2011-12-19

**Authors:** Zsofia Nemoda, Maria Horvat-Gordon, Christine K Fortunato, Emilie K Beltzer, Jessica L Scholl, Douglas A Granger

**Affiliations:** 1 Department of Medical Chemistry, Molecular Biology and Pathobiochemistry, Semmelweis University, Tuzolto utca, Budapest, Hungary; 2 Salimetrics LLC, Innovation Boulevard, State College, Pennsylvania, USA; 3 Department of Human Development and Family Studies and Biobehavioral Health, Pennsylvania State University, Henderson South, University Park, Pennsylvania, USA; 4 Center for Interdisciplinary Salivary Bioscience Research, Johns Hopkins University School of Nursing and Bloomberg School of Public Health, Baltimore, Maryland, USA

## Abstract

**Background:**

Technical advances following the Human Genome Project revealed that high-quality and -quantity DNA may be obtained from whole saliva samples. However, usability of previously collected samples and the effects of environmental conditions on the samples during collection have not been assessed in detail. In five studies we document the effects of sample volume, handling and storage conditions, type of collection device, and oral sampling location, on quantity, quality, and genetic assessment of DNA extracted from cells present in saliva.

**Methods:**

Saliva samples were collected from ten adults in each study. Saliva volumes from .10-1.0 ml, different saliva collection devices, sampling locations in the mouth, room temperature storage, and multiple freeze-thaw cycles were tested. One representative single nucleotide polymorphism (SNP) in the catechol-*0*-methyltransferase gene (COMT rs4680) and one representative variable number of tandem repeats (VNTR) in the serotonin transporter gene (5-HTTLPR: serotonin transporter linked polymorphic region) were selected for genetic analyses.

**Results:**

The smallest tested whole saliva volume of .10 ml yielded, on average, 1.43 ± .77 μg DNA and gave accurate genotype calls in both genetic analyses. The usage of collection devices reduced the amount of DNA extracted from the saliva filtrates compared to the whole saliva sample, as 54-92% of the DNA was retained on the device. An "adhered cell" extraction enabled recovery of this DNA and provided good quality and quantity DNA. The DNA from both the saliva filtrates and the adhered cell recovery provided accurate genotype calls. The effects of storage at room temperature (up to 5 days), repeated freeze-thaw cycles (up to 6 cycles), and oral sampling location on DNA extraction and on genetic analysis from saliva were negligible.

**Conclusions:**

Whole saliva samples with volumes of at least .10 ml were sufficient to extract good quality and quantity DNA. Using 10 ng of DNA per genotyping reaction, the obtained samples can be used for more than one hundred candidate gene assays. When saliva is collected with an absorbent device, most of the nucleic acid content remains in the device, therefore it is advisable to collect the device separately for later genetic analyses.

## Background

In the wake of the Human Genome Project, information from Genome Wide Association (GWA) studies is accumulating at a rapid rate. GWA studies include large numbers of well-characterized cases and several hundred thousand polymorphisms in an attempt to identify candidate genes with plausible linkages to the phenotypes of specific interest [1]. Once identified as biologically plausible, subsequent studies conducted on independent populations endeavour to replicate the genotype-phenotype association, because confirmation of small genetic effect is crucial in complex inheritance disorders and traits. Research groups can potentially use already collected biological samples for genetic analyses. In a series of studies we show that saliva samples, even though originally not designed for genetic analyses, can be reliably used for genotyping genetic polymorphisms. Recommendations are provided to guide researchers with archived specimens, as well as those preparing to launch new data collections.

In studies involving children and healthy subjects, non-invasive sampling of DNA is preferred. Mailing buccal or saliva samples in large-scale epidemiological studies is also the choice of method. Recent studies reveal that high-quality and -quantity DNA can be obtained from saliva samples [2-4]. However, the use of saliva as a biospecimen is associated with several special issues. Depending on the method used to collect saliva, the specimen will yield different volumes, raising the possibility that the quantity of DNA available to be extracted will also vary. Saliva contains a variety of compounds that have the potential to degrade proteins and nucleic acids [5]. If samples are stored or transported at room temperature, the activity of these compounds or their products may affect the DNA extracted from the sample. Even under healthy circumstances, oral fluids contain a diverse array of microbes (e.g., virus, bacteria, and fungi), and therefore estimates of DNA quantity and quality in saliva may be overestimated (or confounded) by DNA from these microorganisms [2]. Cells may also adhere to different devices that are utilized in saliva sample collection (e.g., cotton, foam, and hydrocellulose), causing lower quantities of DNA to be present in the extracted saliva specimen. Additionally, "saliva" is a mixture of different fluids produced by multiple glandular sources (i.e., parotid, sublingual, submandibular salivary glands). Each gland produces a different volume of saliva and secretes different constituents, raising the possibility that specific oral fluid types may contain more or less nucleic acid content. The past two decades have witnessed the adoption and integration of minimally invasive measures (in saliva) in studies of the psychobiology of the stress response (e.g., cortisol, alpha-amylase [6]), secretory products of the endocrine systems (e.g., testosterone [7]), inflammatory processes (e.g., cytokines [8]), and pathogen-specific antibodies (e.g., HSV [9]). Access to these tools is now widespread, collection and assay protocols are being standardized, and salivary analytes and biomarkers are being employed across many subfields.

In a series of comparative analyses of saliva samples collected using commonly employed techniques the effects of saliva volume, handling, and storage conditions, collection device, and sampling location in the mouth were tested on the quantity and quality of extracted DNA. We also confirmed whether the detection of representative single nucleotide polymorphism (SNP) and variable number of tandem repeats (VNTR) are affected by these factors and processes. For the genetic analyses, two functional polymorphisms were selected from the psychogenetic literature [10]. The Val158Met (rs4680) SNP of the catechol-*0*-methyltransferase (COMT) gene causes an amino acid change from valine to methionine at the 158^th ^position in the membrane-bound isoform. The Met-variant results in 2 to 4 times lower enzymatic activity [11], affecting degradation of catecholamines (e.g., dopamine) in the central nervous system. This SNP displays a trimodal distribution of enzyme activity: low (Met/Met), intermediate (Val/Met), and high (Val/Val) [12]. The investigated VNTR was the so-called 5-HTTLPR (serotonin transporter linked polymorphic region) located in the promoter region of the serotonin transporter gene (SLC6A4). It has two main alleles in Caucasians: 14-repeat = Short (S) and 16-repeat = Long (L) allele. The short variant showed reduced transcription activity in a reporter gene system and in lymphoblasts [13,14].

### Study 1: Are the typical volumes of saliva collected in research studies sufficient to enable isolation of DNA for genetic analyses?

The attention saliva has received as a research and diagnostic specimen [15] is largely due to the perception that sample collection is quick and uncomplicated. In many circumstances this claim is true. However, saliva collection from infants less than 3 months of age often results in low specimen volumes [16]. Later in early childhood (12-18 months), saliva collection by a stranger becomes complicated by more frequent occurrences of anxiety and non-compliance with collection procedures [17]. On the opposite end of the continuum, collecting saliva from the elderly can be time-consuming and also may have a high failure rate. Xerostomia (dry mouth) is a common iatrogenic effect of medications, and dry mouth presents in a high percentage of participants in studies of the oldest-old [18]. Thus, in field settings the volume of saliva available to be collected will vary. In Study 1, we tested how variation in the volume of saliva collected affects the quality and quantity of DNA, and the detection of 5-HTTLPR and COMT Val158Met polymorphisms.

### Study 2: Do collection device materials affect the isolation of sufficient quantity and quality DNA from saliva?

Historically, saliva collection devices involve cotton-absorbent materials [19]. When placed in the mouth (2-3 min), cotton saturates with saliva which is then expressed into collection vials by centrifugation or compression [20]. Most of the time, this approach is convenient, simple, and time-efficient. However, when the absorbent capacity is large and sample volume is small, the specimen absorbed can be diffusely distributed and specimen recovery becomes problematic [21]. Contemporary collection methods now employ synthetic or hydrocellulose rather than cotton fibers and yield a much higher sample recovery rate (e.g., hydrocellulose microsponge, synthetic pledget or swab). When the participant is older than 6 years, awake, compliant, and capable of following instructions, collecting whole saliva by passive drool is optimal [17].

It is possible that some of the materials used to collect saliva may bind and retain cells or nucleic acids such that the DNA extracted from saliva using these tools would be compromised. In Study 2, we examined this possibility utilizing four common saliva collection methods (1) BD hydrocellulose microsponge, (2) Richmond cotton rope, (3) Sarstedt synthetic pledget, and (4) Salimetrics synthetic swab, and (5) passive drool as a control.

### Study 3: What are the effects of storing saliva samples at room temperature on the extraction and analysis of DNA?

In research studies, saliva specimens are often gathered in the field or in such conditions that restrict the way in which they can be handled and stored. Typically, once a specimen is collected, samples are kept cold and then frozen to maintain sample integrity. Refrigeration prevents degradation of some salivary analytes and restricts proteolytic enzyme activity and bacteria growth. For large-scale national surveys, in which investigators are working in remote areas [22,23] or for diurnal cortisol assessment where patients are collecting samples at home [24], freezing and shipping samples frozen can be cost-prohibitive. Appropriate sample handling after collection is essential to maintaining sample integrity; the handling plan needs to be worked out *a priori *and matched specifically to the "needs" of the analytes to be measured. For example, salivary cortisol or alpha-amylase levels were shown to be stable at room temperature (RT) for up to 5 days [25,26]. DNA is very stable molecule; however, with respect to extracting DNA from saliva held at RT for any length of time, there are two issues. First, when measuring the quantity and quality of DNA spectrophotometrically, the measurement technique does not differentiate between human and microbial DNA. Bacteria load and growth in specimens held at RT raise the possibility that the amount of microbial DNA in the extracted DNA is rising over time in proportion to bacteria growth. Second, bacterial DNAses can degrade human DNA, raising the possibility that saliva samples held at RT for varying lengths of time would yield lower quantities of human DNA suitable for determination of polymorphisms. In Study 3, saliva samples were held at RT for up to 5 days so the effects of this sample treatment on DNA quality, quantity, and genotyping could be observed.

### Study 4: Do archived saliva samples that have been exposed to multiple freeze-thaw cycles yield sufficient quantity and quality DNA for genetic analyses?

Saliva samples that have already been assayed for multiple analytes and archived have often been subjected to multiple freeze-thaw cycles. Levels of certain salivary steroid hormones (e.g., cortisol, progesterone) and protein biomarkers (e.g., alpha-amylase, immunoglobulin A) show stability over the repeated freeze-thaw cycles in pilot studies [25-28]. However, the extraction of DNA from saliva may be decreased due to freeze-thaw cycle-related damage to DNA or enhanced by the effects of freeze-thaw cycles on the breakdown of cellular materials. Therefore, saliva samples that have been archived after multiple freeze-thaw cycles may yield different quantity and quality DNA. In Study 4, we explore this possibility.

### Study 5: When oral fluids are collected from specific areas in the mouth associated with different salivary glands, is there a difference in DNA quality or quantity?

Whole saliva is a composite derived from oral fluids secreted by many salivary glands. The largest glands are the parotid (located upper posterior area of oral cavity), submandibular (lower area between cheek and jaw), and sublingual (under tongue). Some oral fluid also comes from the serum via the crevicular fluid (area between teeth and gums) or via mucosal damage and by leakage. Each type of gland secretes fluid with characteristic composition and properties [29]. The contribution each source gland makes to the overall "oral fluid pool" is variable; consequently, the composition of the saliva exhibits considerable variation. For instance, mucins make saliva viscous, elastic, and sticky to protect tooth enamel against wear and encapsulate microorganisms. These glycoproteins are not present in oral fluid secreted by the parotid gland. Under resting conditions, minimal fluid contribution from the parotid gland occurs and the levels of mucins in saliva will be high (consequentially, specimens will be more viscous). However, after stimulation, saliva flow from the parotid gland (e.g., in response to autonomic nervous system activation) will dilute the concentration of mucins and specimens will be less viscous [29].

The concentration of some analytes of interest is higher in the output of some salivary glands than others. As different salivary glands contribute different amounts to oral fluids, results may differ depending on where in the mouth samples are collected. These observations raise the possibility that the unsystematic application of common saliva collection techniques might cause researchers to inadvertently sample oral fluids from different areas in the mouth, and that this variation in oral fluid type may contribute measurement error. This phenomenon seems less problematic when the specimen collected is whole saliva but more likely when oral fluid specimens are collected with absorbent materials such as filter papers, microsponges, or small oral swabs that may not be placed in the same specific area in the mouth [30] consistently within and/or across sampling occasions [31,32]. In Study 5, we explored the contribution of oral fluid type (i.e., sampling saliva from different locations in the mouth), to differences in the quantity and quality of DNA extracted from those biospecimens.

## Methods

### Participants and Design

The following five studies were planned in compliance with the guidelines of the Helsinki Declaration. The protocol and procedures involving healthy participants were reviewed and approved by the Penn State University institutional review board, and informed consent was obtained from all participants.

Study 1: Ten healthy adults donated a bolus sample (2 × 2 ml) of whole saliva over 10 minutes. Following the recommendations of Granger and colleagues [17], samples were collected using the passive drool technique. Briefly, participants were asked to imagine that they were chewing their favourite food, move their jaws as if they were chewing that food, and gently force the pooling saliva generated through a short plastic drinking straw into a polypropylene collection vial (2.0 ml). After collection, samples were vortexed (mixed) and aliquoted into separate cyrogenic storage vials in volumes of .10, .25, .50, and 1.0 ml. Samples (*n *= 40) were stored frozen at -20°C until extraction and assay.

Study 2: Ten healthy adults donated a bolus sample (5 ml) of whole saliva. After collection, samples were vortexed (mixed) and aliquoted into five separate 2 ml centrifuge tubes in volumes of .50 ml. A different collection device (1) BD hydrocellulose microsponge (BD Medical-Ophtalmic Systems, Waltham, MA, USA), (2) Richmond cotton rope (Richmond Dental, Charlotte, NC, USA), (3) Sarstedt synthetic pledget (Sarstedt AG & Co, Nümbrecht, Germany), and (4) Salimetrics synthetic swab (Salimetrics LLC, State College, PA, USA) was submerged into each aliquot for 3 minutes. Saliva was expressed from the devices by centrifugation (1500 g) for 15 min. Saliva samples (*n *= 50) were stored frozen at -20°C until the day of extraction.

Study 3: Ten healthy adults donated a bolus sample (3 ml) of whole saliva. After collection, samples were vortexed (mixed) and aliquoted into five separate volumes of .50 ml. One aliquot was frozen at -20°C immediately thereafter and served as an "untreated" control. The remaining four aliquots were held at room temperature (RT, 18-22°C) and frozen after 24 hrs, 48 hrs, 72 hrs, or 120 hrs (5 days). At the conclusion of the 5-day period, all samples (*n *= 50) remained stored frozen at -20°C until the day of extraction.

Study 4: Ten healthy adults donated a bolus sample (3 ml) of whole saliva by the passive drool technique. After collection, samples were vortexed (mixed) and aliquoted into five separate volumes of .50 ml in 2.0 ml cryogenic vials. One aliquot was extracted immediately without freezing and served as an "untreated" control. The remaining 4 aliquots were exposed to 1, 2, 4 or 6 freeze-thaw cycles. The duration of each "freeze" cycle was 45 min at -20°C and each "thaw" cycle was 45 min at RT. On the final freeze-thaw cycle for each condition, samples were left frozen at -20°C until the day of extraction.

Study 5: Ten healthy adults first donated .50 ml of whole saliva by the passive drool technique into a 2 ml vial. Then, each participant placed hydrocellulose microsponges (BD Medical-Ophtalmic Systems, Waltham, MA, USA) *simultaneously *in three different areas of the mouth for 5 min. Devices were placed under the tongue to absorb oral fluid produced in the sublingual salivary gland area, between the lower left cheek and gum to collect oral fluid from the submandibular salivary gland area, and also between the upper right cheek and gum in the rear of the mouth by the jaw hinge to gather oral fluid from the parotid salivary gland area. Upon removal from the mouth, participants sealed the devices in a 2 ml cyrovial, and the samples were stored at -80°C until assay. After a 10 min wait period, the same participants placed 1 × 4 cm absorbent synthetic oral swabs (Salimetrics LLC, State College, PA, USA) *simultaneously *in the three different areas of the mouth listed above for 5 min. Upon removal, each swab was sealed in a polypropylene carrier tube and stored at -80°C until assay. The order of device use (i.e., microsponge and then oral swab, or visa versa) was counterbalanced among participants. Sample volume recovery was measured when transferring sample into extraction tube. Saliva samples (*n *= 70) were stored frozen at -20°C until the day of extraction.

### DNA Extraction

A modified Puregene (Gentra) DNA isolation kit was used for DNA extraction. On the day of extraction, all samples were thawed and then centrifuged at 2300 g for 10 minutes. After centrifugation of the saliva, the top supernatant was removed by pipette and discarded. Cell lysis was achieved by adding 350 μl of lysate solution with .9 U proteinase K to the precipitated cells (pellet). The cell lysate was then incubated at 56°C with brief periods of mixing for 1 hour. To precipitate proteins, the sample was cooled to RT and 100 μl of protein precipitation solution was added to the lysate. The mixture was placed in an ice bath for 5 min and then centrifuged at 15,000 g for 3 min. DNA extraction was achieved by pipetting the supernatant into a 1.5 ml microcentrifuge tube containing 350 μl 100% isopropanol with .10 mg glycogen. The solution was mixed via several gentle inversions and allowed to sit undisturbed for 5 min at RT before centrifuging it at 15,000 g for 5 min. Afterwards the supernatant was discarded without disturbing the DNA-containing pellet. The DNA pellet was washed two times in 350 μl 70% ethanol by gently inverting the tube and centrifugation at 15,000 g for 5 min. After the final wash, all ethanol was removed from the sample and the DNA was allowed to dry for 15 min at RT. DNA was then dissolved by adding 50 μl warm TE (10 mM Tris, 1 mM EDTA) buffer (for extended storage), and samples were stored at -20°C. DNA quality and quantity were estimated by measuring the absorbance of the sample at wavelengths 260 nm and 280 nm using a spectrophotometer (NanoVue Spectrophotometer, GE Healthcare). Nucleic acid absorbs ultraviolet light (UV) with an absorption peak at 260 nm, whereas proteins with aromatic side chains have an absorption peak at 280 nm. An optical density of 1 at 260 nm corresponds to about 50 ng/μl of double-stranded DNA, whereas the 1.8 or higher 260/280 nm ratio indicates that the DNA sample is relatively free from protein contamination (the acceptable range is 1.5-2.0). After the NanoVue measurements, the DNA samples were diluted to a final concentration of 15 ± 5 ng/μl for the genetic analyses. DNA samples were stored frozen until the day of assay.

After extracting and measuring the DNA recovered in Study 2, it was hypothesized that a large percentage of the samples' nucleic acid content was retained in the absorbent devices. An "adhered cell" modified extraction technique was developed in effort to recover the remaining DNA in the collection devices. The "dry" collection devices, which had been re-frozen at -20°C following the centrifugation step as presented above, were then retrieved and used for subsequent analyses. The DNA in the collection devices was recovered by adding the 350 μl of lysate solution (with .9 U proteinase K) onto the device. After 1 hour of incubation at 56°C the collection device was centrifuged and the resulting filtrate solution used to complete the extraction process, taking up the procedure at the protein precipitation step and completing as described previously.

### Genotyping 5-HTTLPR

Polymerase chain reaction (PCR) was used according to Wendland et al. [33] with the HotStar Taq DNA-polymerase kit (Qiagen) applying .25 U enzyme in a total volume of 10 μl, and 10-20 ng genomic DNA. Thermocycling was initiated by a 15 min 95°C denaturation step, followed by 35 cycles of 94°C 1 min, 65°C 30 sec, 72°C 1 min, and a 10 min 72°C final extension (Perkin Elmer 9700 Thermal Cycler). The VNTR was determined by capillary electrophoresis using a 3130xl Genetic Analyzer to separate the 469 bp PCR product of the S allele from the 512 bp PCR product of the L allele. To prepare for the electrophoresis analysis, 1 μl PCR product was dissolved in formamide loading solution with GeneScan Rox size standard in a total volume of 10 μl and heated at 95°C for 5 min, followed by at least 1 min at -20°C. For data analysis, GeneMarker^® ^HID software was used; results are reported for the 5-HTTLPR as S/S, S/L, and L/L genotype groups. The genotyping procedure was carried out at the Nucleic Acid and Protein Core Facility of the Children's Hospital of Philadelphia Research Institute with lab staff blind to the sample data.

### Genotyping COMT Val158Met

A 5' nuclease assay was used for the COMT Val158Met SNP (rs4680) with a pre-designed TaqMan kit (C_25746809_50, Applied BioSystems) and 10-20 ng of genomic DNA. The amplification conditions were: 2 min at 50°C and 10 min at 95°C followed by 40 cycles of 15 sec at 92°C and 90 sec at 60°C in ABI Prism^® ^7500 (Applied Biosystems). Allelic discrimination analysis was performed using the Sequence Detector Systems software; results are reported in three COMT genotype groups: Met/Met, Val/Met, Val/Val. The genotyping procedure was performed at the Mitotyping Technologies Laboratory (State College, PA) with lab staff blind to the sample descriptions.

In each study the reference genotypes of the participants were obtained from the .50 ml whole saliva samples collected by the passive drool technique and stored under the control condition, i.e., frozen within half an hour of the collection and thawed only once on the day of DNA extraction.

## Results and Discussion

### Study 1

In the first study comparing different volume of whole saliva one-way repeated measure ANOVAs were computed with sample volume (.10, .25, .50, and 1.0 ml) as a 4-level independent variable. DNA concentration (ng/μl) and the 260/280 nm ratio were the dependent measures. An outlier greater than 3 *SD *from the mean was removed from the 1.0 ml condition. Table 1 contains the means and standard deviations for the DNA quantity and quality at each sample volume. A linear contrast was computed followed by *a priori *comparisons (t-tests) to decompose the nature of the effect of sample volume.

**Table 1:** Study 1: Means (Standard Deviations) of DNA sample characteristics isolated from different volumes of whole saliva

	**Sample Volume**			
	**.10 ml**	**.25 ml**	**.50 ml**	**1.0 ml**
DNA Concentration (ng/μl)	28.25 (15.36)	68.00 (39.66)	116.90 (55.19)	239.78 (144.27)
DNA Quality (260/280 nm)	1.78 (.10)	1.75 (.10)	1.74 (.14)	1.79 (.11)
DNA Total Amount (μg)	1.43 (.77)	3.40 (1.98)	6.24 (3.38)	11.99 (5.71)

Note: At the end of DNA isolation all samples were dissolved in 50 μl final volume, and 2 μl of these samples were used for DNA concentration and 260/280 nm ratio measurements by NanoVue Spectrophotometer. For a better comparison with other studies, the total amount of DNA is also provided in the table.

#### DNA Quantity and Quality

Results revealed significant effects of sample volume on DNA quantity, *F *(3, 24) = 31.36, *p *<.001. The linear contrast was significant, *F *(1, 9) = 7.02, *p *< .05. *A priori *comparisons (t-tests, one-tailed) revealed that DNA concentration was lower in the .10 ml sample than in the .25 ml sample, *t *(9) = 4.99, *p *< .001; lower in the .25 ml sample than in the .50 ml sample, *t *(9) = 3.42, *p *< .01; and lower in the .50 ml sample than in 1.0 ml, *t *(8) = 6.45, *p *< .001. By contrast, the main effect of volume on DNA quality was not significant, *F *(3, 24) = 1.58, *ns*. As can be seen in Table 1, even with only .10 ml of whole saliva in the specimen, on average, the DNA extracted exceeded 20 ng/μl concentration (1 μg amount) and the quality of the specimen was acceptable.

#### Genotyping

Among the 10 subjects, all 3 genotypes were represented at both polymorphisms: COMT Met/Met (*n *= 3), Val/Met (*n *= 5), Val/Val (*n *= 2); 5-HTTLPR S/S (*n *= 1), S/L (*n *= 6), L/L (*n *= 3). At the COMT SNP, the genotyping success rate of the first run (using 10-20 ng DNA) were the following: all samples from the .10 ml and 1.0 ml conditions, 8/10 of the .25 ml samples, and 9/10 of the .50 ml samples were genotyped successfully in the first run. The 3 samples which needed repetition were successfully genotyped in the second run using 40-60 ng DNA. For 5-HTTLPR, the rate of successful identification was 100% in the first genotyping run.

Whole saliva samples with volumes of *at least *100 μl were sufficient to extract good quantity and quality DNA to make accurate determinations of genotypes for representative SNP and VNTR polymorphisms. Based on sensitive genotyping methods using 10 ng DNA per polymorphism, the DNA samples obtained from 100 μl of whole saliva are sufficient - on average - for genotyping more than 100 candidate polymorphisms. However, we have to note that the lowest DNA concentration was 9.9 ng/μl (total DNA amount .50 μg) among the 100 μl samples, hence this sample can be used only for 25-50 genotyping reactions. The total DNA amount isolated from *at least *250 μl of whole saliva can be sufficient for whole genome analyses requiring 1.5-3 μg DNA. However, because of the variance among samples (the mean DNA amount was 3.4 μg at the 250 μl samples, but ranging from .9 μg to 7.4 μg), we suggest to collect at least 500 μl (.50 ml) of whole saliva for GWA studies (the mean DNA amount was 6.2 μg at the 500 μl samples, ranging from 1.5 μg to 12.4 μg).

### Study 2

In the second study assessing the effects of different collection devices the volume recoveries - as measured when transferring the saliva filtrate to the extraction tube - were, on average, .36 ml (*SD *= .03) for hydrocellulose microsponge, .35 ml (*SD *= .03) for the cotton rope, .39 ml (*SD *= .12) for the synthetic pledget; and .42 ml (*SD *= .02) for the synthetic swab. One-way repeated measure ANOVAs were computed with collection device type as a 5-level independent variable separately for DNA concentration (ng/μl) and 260/280 nm ratio. Planned *a priori *comparisons (t-tests) tested the difference between whole saliva (control) and each individual device.

#### DNA Quantity

There was a main effect of collection device/method on DNA concentration, *F *(4, 36) = 15.83, *p *< .0001. On average, the concentration of DNA extracted from whole saliva (*M *= 256.2 ng/μl, *SD *= 188.24) was significantly higher than the average level extracted from saliva collected with the microsponge (*M *= 10.30 ng/μl, *SD *= 6.11), *t *(9) = 4.17, *p *< .01; than the cotton rope (*M *= 8.35 ng/μl, *SD *= 3.62), *t *(9) = 4.21, *p *< .01; than the pledget (*M *= 12.80 ng/μl, *SD *= 7.94), *t *(9) = 4.23, *p *< . 01; and the synthetic swab (*M *= 53.41 ng/μl, *SD *= 49.20), *t *(9) = 3.47, *p *< .01. The levels obtained among the specific absorbent devices (hydrocellulose microsponge, cotton rope, pledget, and synthetic swabs) did not significantly differ from one another. Also, there was no evidence that sample volume recovered from the devices was associated with the concentration of the DNA extracted.

#### DNA Quality

In the saliva filtrate there was also a main effect on the 260/280 nm ratio, *F *(4,36) = 7.02, *p *< .0001. The average 260/280 nm ratio in sample extracted from whole saliva (*M *= 1.75, *SD *= .16) was significantly higher than from the hydrocellulose microsponge filtrate (*M *= 1.60, *SD *= .17), *t *(9) = 3.50, *p *< .01. The pledget filtrate yielded the highest quality DNA (*M *= 1.88, *SD *= .09, *t *(9) = -2.20, *ns*), whereas the average 260/280 nm ratio of filtrates from cotton rope (*M *= 1.69, *SD *= .11, *t *(9) = .96, *ns*) and synthetic swab (*M *= 1.72, *SD *= .16, *t *(9) = 1.63, *ns*) were similar to that of the whole saliva.

#### Genotyping

Genotype groupings at the two polymorphisms were: COMT: Val/Met (*n *= 6), Val/Val (*n *= 4); 5-HTTLPR: S/S (*n *= 3), S/L (*n *= 4), L/L (*n *= 3). Despite the significant differences in DNA quantity by collection method, there was 100% accuracy in the determination of COMT Val158Met polymorphism within each subject across collection device type, and there was only one misgenotyping at the 5-HTTLPR (S/L instead of L/L). All samples except one from the pledget filtrates yielded good genotype calls in the first genotyping run at the COMT SNP; the sample which needed repetition was successfully genotyped in the second run. For 5-HTTLPR, the genotyping success rate of the first genotyping run was 100% for whole saliva, cotton rope and pledget filtrate samples. There was one non-determination for the microsponge filtrates, and the misgenotyping mentioned above occurred with one of the synthetic swab filtrates.

As can be seen in Table 2, after extracting DNA from the saliva passed through the devices (i.e., the saliva filtrate), the quantity of DNA recovered was very low compared to the whole saliva sample control. The "adhered cell" hypothesis was confirmed by the results of the modified extraction, as the quantity and quality of the DNA samples extracted from the adhered cells were comparable to that of the whole saliva samples (Table 2). More notably, all of these samples could be accurately genotyped for the COMT SNP in the first genotyping run. For each specimen, a percentage of DNA extracted from the collection device (i.e., adhered cell) was computed. On average, the percentage of DNA retained in each device was 91.34% for the hydrocellulose microsponge, 92.32% for the cotton rope, 86.74% for the pledget, and 53.91% for the synthetic swab. Table 2 presents the descriptive data for the DNA extracted via the *Saliva Filtrate *and *Adhered Cell *procedures.

**Table 2:** Study 2: Means (Standard Deviations) of DNA sample characteristics obtained comparing different collection devices and passive drool (control)

Device Type:	**Microsponge**		**Cotton Rope**		**Synthetic Swab**		**Pledget**		**Passive **
	**SF**	**AC**	**SF**	**AC**	**SF**	**AC**	**SF**	**AC**	**Drool**
DNA Concentration (ng/μl)	10.30	176.20	8.35	155.35	53.41	65.38	12.28	125.70	262.68
	(6.11)	(107.03)	(3.62)	(104.60)	(49.20)	(43.56)	(7.94)	(122.68)	(179.00)
DNA Quality (260/280 nm)	1.60	1.93	1.69	1.87	1.72	1.90	1.88	1.84	1.75
	(.17)	(.17)	(.11)	(.14)	(.16)	(.14)	(.09)	(.21)	(.16)

Note: SF = Saliva Filtrate; AC = Adhered Cell; Passive Drool = .50 ml of whole saliva.

When saliva is collected using absorbent devices, a considerable portion of the nucleic acid content is retained in the absorbent devices. The DNA retained in collection devices studied here can be obtained using a secondary extraction protocol. Despite this phenomenon, DNA extracted from saliva filtrate was sufficient to accurately call the COMT Val158Met polymorphism in every condition, and detection of the 5-HTTLPR in almost every condition.

### Study 3

In the third study analyzing the effect of room temperature storage one-way repeated measure ANOVAs were computed with time stored at RT prior to freezing as a 5-level independent variable separately for DNA concentration and 260/280 nm ratio. A planned comparison tested the linear effect of time, and *a priori *contrasts (t-tests) compared the control condition (frozen immediately) to each individual time point (24, 48, 72, and 120 hrs).

#### DNA Quantity and Quality

**Figure 1:**
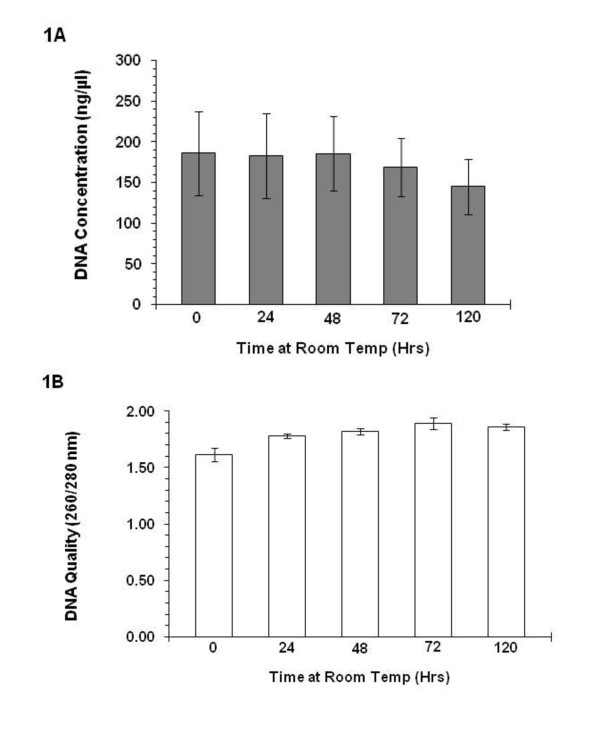
**Study 3 "Time Left at Room Temperature" concentration (Figure 1A) and quality (Figure 1B) measures of salivary DNA obtained from passive drool**. Saliva samples left out at room temperature for 0, 24, 48, 72, and 120 hours. Note: .50 ml of whole saliva was used in each condition. Error bars represent the standard error.

The main effect of storage at RT on DNA concentration was not significant, *F *(4, 36) = .94, *ns*. By contrast, there was a significant main effect of time at RT on the 260/280 nm ratio, *F *(4, 36) = 11.40, *p *< .0001 (see Figure 1). On average, the control condition (frozen immediately, no storage at RT) had a lower 260/280 nm ratio (*M *= 1.61, *SD *= .19) than samples stored at RT for 24 hrs (*M *= 1.78, *SD *= .07), 48 hrs (*M *= 1.82, *SD *= .09), 72 hrs (*M *= 1.89, *SD *= .17), or 120 hrs (*M *= 1.86, *SD *= .09), *t*s (9) = 3.34 to 4.08, *p*s < .01. The linear contrast was significant, *F *(1, 9) = 16.59, *p *< .01, indicating that as hours at RT increased, the 260/280 nm ratio tended to increase. *A priori *comparisons (t-tests, one-tailed) revealed the RT-related increases in the 260/280 nm ratio were more pronounced between the control and 24 hrs, (*t *(9) = 3.33, *p *< .01), and between the 24 hrs and 48 hrs, (*t *(9) = 2.15, *p *< .05) time points, and less pronounced between the 48 hrs to 72 hrs (*t *(9) = 1.93, *p *< .05), and between 72 hrs to 120 hrs (*t *(9) = 1.01, *ns*) intervals.

#### Genotyping

The genotypes at the studied polymorphisms were: COMT Met/Met (*n *= 2), Val/Met (*n *= 5), Val/Val (*n *= 3); 5-HTTLPR S/S (*n *= 1), S/L (n = 5), L/L (*n *= 4). The genotyping success rate of the first run was 100% for the 5-HTTLPR, whereas 7 samples out of the 50 needed repetition at the COMT SNP (successfully determined sample ratios in the first genotyping run were: frozen immediately: 8/10; frozen 24 hrs: 8/10; frozen 48 hrs: 8/10; frozen 72 hrs: 10/10; frozen 120 hrs: 9/10). All samples yielded accurate genotype calls in the second run.

Consistent with the literature documenting the stability of nucleic acids, we observed that saliva samples could be held at RT for many days without compromising the usability to analyze candidate gene polymorphisms. Storage of saliva samples at RT prior to freezing, similar to sending samples through the mail unrefrigerated, for up to 5 days did not influence the accuracy of COMT Val158Met or 5-HTTLPR genotyping. The success rate of the first genotyping run was not associated with the time samples were held at RT, and probably reflects technical issues (e.g., pipetting problem at the PCR).

### Study 4

In the fourth study assessing the effect of multiple freeze-thaw cycles one-way repeated measure ANOVAs were computed with number of freeze-thaw cycles as a 5-level independent variable separately for DNA concentration and 260/280 nm ratio. A planned comparison tested the linear effect, and *a priori *comparisons (t-tests) compared the no freeze-thaw cycle condition to each exposure condition (1, 2, 4, and 6 freeze-thaw cycles).

#### DNA Quantity and Quality

**Figure 2:**
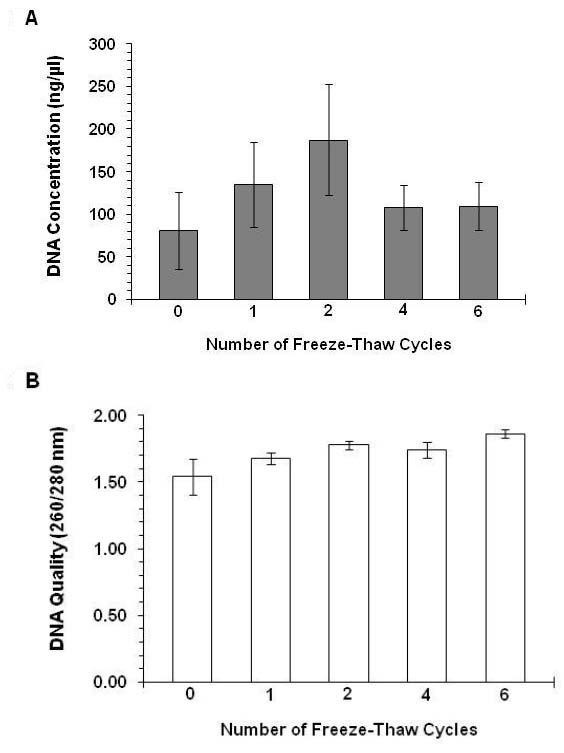
**Study 4 "Freeze-Thaw Effects" concentration (Figure 2A) and quality (Figure 2B) measures of salivary DNA obtained from passive drool**. Saliva samples exposed to 0, 1, 2, 4, and 6 freeze-thaw cycles. Note: .50 ml of whole saliva was used in each condition. Error bars represent the standard error.

Freeze-thaw conditions had a main effect on both DNA concentration, *F *(4, 36) = 2.86, *p *< .05 and DNA quality, *F *(4, 36) = 3.30, *p *< .05. However, the linear contrast for DNA quantity was not significant; the main difference came from the lower DNA concentration of the extracted immediately samples compared to the samples exposed to one or two freeze-thaw cycles (but not to the 4 or 6 freeze-thaw cycles samples, see Figure 2A). In terms of DNA quality, the linear contrast was significant, *F *(1, 9) = 5.39, *p *< .05. On average, the 260/280 nm ratio was comparable in samples extracted immediately without a freeze-thaw cycle (*M *= 1.54, *SD *= .42), and in samples exposed to a single freeze-thaw cycle (*M *= 1.67, *SD *= .14), *t *(9) = 1.13, *ns*. Samples with one or fewer freeze-thaw cycle exposure yielded lower 260/280 nm ratio on average (*M *= 1.61, *SD *= .25) than samples exposed to 2 or more freeze-thaw cycles (*M *= 1.79, *SD *= .07), *t *(9) = 2.47, *p *< .05. DNA quality was not statistically different in samples exposed to 2, 4, or 6 freeze-thaw cycles (see Figure 2B).

#### Genotyping

All 3 genotypes were represented at both polymorphisms: COMT Met/Met (*n *= 1), Val/Met (*n *= 6), Val/Val (*n *= 3); 5-HTTLPR S/S (*n *= 5), S/L (*n *= 3), L/L (*n *= 2). The determination of 5-HTTLPR and COMT Val158Met polymorphism was not affected by the number of freeze-thaw cycles or by the 260/280 nm ratio values; all samples from the same individual gave identical results. The genotyping success rate of the first run was 100% at the 5-HTTLPR, whereas 6 samples out of the 50 needed repetition at the COMT SNP (all the control, 4 and 6 freeze-thaw cycle samples gave good quality signals in the first run but 30% of the 1 and the 2 freeze-thaw cycle samples did not, these samples were successfully genotyped in the second run).

The effects of freeze-thaw cycles on saliva samples were more pronounced for the quality of DNA extracted from archived specimens. On average, the ratio of DNA to large molecule contaminants was enhanced by freezing and thawing the sample. The lower 260/280 nm ratio and the lower DNA yield of the samples extracted immediately compared to that of the frozen samples were probably due to the pellet formation after centrifugation, because many times the pellets in unfrozen saliva were very loose, and the supernatant was very viscous and thick. As a result of incomplete separation during centrifugation, some of the cell fraction from the unfrozen samples was lost when the samples were decanted. However, the results of the genetic analyses were unaffected (all the extracted immediately samples were genotyped successfully in the first run). Overall, neither the quantity of DNA extracted nor the genotype determinations were systematically influenced by exposure to freeze-thaw conditions.

### Study 5

In the last study comparing sampling locations in the mouth the volume recoveries for the microsponge condition were, on average: sublingual .26 ml (*SD *= .08), parotid .19 ml (*SD *= .11), and submandibular .16 ml (*SD *= .13). For the synthetic swab volume recoveries were, on average: sublingual .92 ml (*SD *= .47), parotid .49 ml (*SD *= .27), and submandibular .67 ml (*SD *= .44). A series of 2 (hydrocellulose microsponge vs. synthetic swab) by 3 (sampling location: sublingual, parotid, submandibular) repeated measures ANOVAs were computed separately for DNA concentration and the 260/280 nm ratio.

#### DNA Quantity and Quality

**Figure 3:**
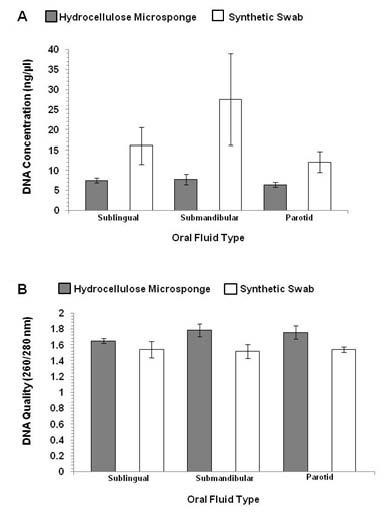
**Study 5 "Sampling Location" concentration (Figure 3A) and quality (Figure 3B) measures of salivary DNA collected from three oral locales**. Saliva samples collected from sublingual, submandibular, and parotid oral glands using hydrocellulose microsponge and synthetic swab collection devices. Error bars represent the standard error.

The effect of device type on DNA concentration of the saliva filtrates was not significant, *F *(1, 9) = 3.64, *ns*, but there was a main effect of device type on DNA quality, *F *(1, 9) = 6.85, *p *< .05 (Figure 3). Given the findings for Study 2, this redundant observation is not described in any further detail.

#### Genotyping

All 3 genotypes were represented at both polymorphisms: COMT Met/Met (*n *= 2), Val/Met (*n *= 4), Val/Val (*n *= 4); 5-HTTLPR S/S (*n *= 2), S/L (*n *= 5), L/L (*n *= 3). The genotyping success rate was 98.6% for both polymorphisms, with one sample out of 70 giving no result.

The findings of collection device effectiveness at different selected oral sampling locales are consistent with the findings in Study 2 regarding collection devices. That is, Study 5 confirms that the synthetic swab gives a greater recovery of DNA from the saliva filtrate than the microsponge. This could be directly related to the fact that the synthetic swab collects a larger amount of oral fluid which can then be used for extraction. The collection devices for Study 5 were not extracted using the "adhered cell" extraction developed for Study 2 due to the similarity of the observations for the initial extraction noted. As expected, oral sampling location does not significantly affect the quantity or quality of DNA recovered (per each device evaluated). As in the previous studies, the quantity of DNA which was recovered is still sufficient to perform multiple genotyping procedures. Genotyping of both the COMT rs4680 and 5-HTTLPR VNTR polymorphisms was unaffected by the study conditions.

## Conclusions

To summarize, these experiments revealed several noteworthy findings. First, whole saliva samples with volumes of *at least *100 μl were sufficient to extract good quality and quantity DNA. Second, when saliva is collected via absorbent devices, the majority of the nucleic acid content remains in the device and is not expressed in the saliva filtrate. It is important to note that the nucleic acid retained in these devices can be recovered with a follow-up extraction procedure. Third, the effects of saliva storage at room temperature (for up to 5 days), repeated freeze-thaw cycles (up to 6 cycles), and sampling different oral fluid types on DNA extraction from saliva were negligible. Finally, across the conditions studied, the DNA extracted from saliva samples was sufficient to consistently determine genotypes for representative SNP (COMT Val158Met) and VNTR (5-HTTLPR) polymorphisms. Since PCR-based candidate gene analyses usually require 5-10 ng of DNA per polymorphism, the lowest DNA concentration samples could be still used for determination of 20-50 polymorphisms. Collectively, these observations serve to raise awareness in the scientific community that a number of genetic polymorphisms can be determined from saliva specimens that have already been assayed and stored at -20 or -80°C. To do so, of course, would require appropriate ethical review and approval. Also, the cell pellet of the same saliva sample can be used for genetic analyses in parallel to salivary biomarker analysis conducted from the supernatant, reducing the cost and time of collecting biological specimen from the study participants.

Our main goal was to show that sufficient amount of DNA can be obtained from saliva samples without immediate addition of cell lysis buffer, which is used by the most widespread saliva DNA collection techniques, opening up the possibilities to use saliva samples for genetic analyses without interfering with hormone, cytokine or other biomarker analyses. When comparing the average amount of DNA per 1 ml saliva calculated from the samples we stored under the control condition (i.e., frozen within half an hour of the collection) to published data of other research groups using whole saliva mixed with cell lysis buffer at the time of collection, our results (ranging from 11.99 μg/ml to 26.67 μg/ml) represented the lower end of the range, as the reported average DNA amounts in pilot studies vary from 11.4 μg/ml [2] to 77.4 μg/ml [3], whereas in large scale studies the calculated total DNA per 1 ml saliva is between 23-42 μg/ml [4,34,35]. It is interesting to note that the total amount of DNA obtained from saliva samples shows huge variability both under experimental laboratory settings (e.g., 155 ± 103 μg per 2 ml saliva [3]) and in epidemiological studies (e.g., 92 ± 74 μg per 4 ml saliva from a sample of 555 adults [34]).

Investigators interested in the possibility of including DNA analyses in current or future projects, who are using absorbent materials (sponges, pledgets, swabs, ropes) to collect saliva specimens, should be warned that they are discarding the majority of the cells needed to extract nucleic acids when they remove the saliva filtrate and discard the collection device into biohazard waste. Given the expense of sample collection, particularly in high-risk or special populations, investigators may wish to consider storing the collection devices (and the cell fraction contained therein) to extract DNA from it.

Researchers should be pleased to know that DNA can be obtained from simple and cost-effective collection techniques already employed in the field. That is, even if specimens have not been obtained using devices specific for the collection, stabilization, and transportation of DNA, they can be still used for determination of candidate gene polymorphisms using sensitive genotyping methods. Sufficient quality and quantity DNA can be recovered even from very small samples of saliva, despite thawing and refreezing as part of testing for other analytes, and even if they have been shipped or stored unrefrigerated for (up to 5) days. The success rates of the first genotyping rounds (using 10-20 ng DNA) were 94-98% across the studies at the two representative candidate polymorphisms, with only one sample out of the 260 giving no results at either polymorphisms, suggesting that the DNA isolation was unsuccessful only in .38% of samples. All samples retested with higher amounts of DNA (40-60 ng) could be genotyped with repeated measurements (see results for COMT SNP), hence the final success rate reached 99.6%.

This series of studies has several methodological strengths which lead us to have confidence in the conclusions reached. However, it is important to note that the conditions under which these specimens were collected are different from the field conditions in which actual specimens would be collected. Therefore, the findings illustrate potential, rather than actual, pitfalls and problems that might be associated with extracting DNA from saliva. Studies in field settings aimed to corroborate these results would be a worthwhile next step. Another important limitation of this study is that only spectrophotometric measurement was used to determine DNA concentration, preventing differentiation between human and bacterial DNA. Therefore, the DNA concentration values of samples left out at room temperatures for days probably do not reflect the true amounts of human DNA. However, the genetic analyses of these (probably contaminated) samples yielded the same genotypes as those of the control samples (frozen immediately), suggesting that sensitive PCR-methods using human specific primers and/or probes are able to detect human genetic variations accurately even from a small fraction of human DNA. Also, we limited our scope to a single representative SNP and VNTR. Future studies should consider expanding the range of candidate polymorphisms to evaluate the breadth of these observations.

In this decade, an initiative led by the U.S. National Institute of Craniofacial and Dental Research (NICDR) characterized the salivary proteome. This ground-breaking effort revealed that as many as 2,000 analytes of potential interest to the behavioral, health, sports, and developmental sciences are present in oral fluids [36]. This library of potential salivary analytes in combination with genetic polymorphisms, offer scientists an opportunity to take the lead by testing models that involve multi-level components of biological systems and their relationship to behavior, health, and social forces. As with all major developments, however, many questions remain unanswered and specialized issues exist that require empirical attention. Drawing attention to these issues will increase the probability that DNA, in combination with other salivary analytes, will be more successfully integrated into the next generation of studies, and in doing so, will set a more solid foundation for the eventual translation of the basic findings into screening, prevention, clinical investigation, and diagnostics.

## Competing interests

The studies reported in this paper were conducted while Dr. Granger was on the Faculty in the Department of Biobehavioral Health at Penn State University. In the interest of full disclosure, Douglas A. Granger is Chief Scientific and Strategy Advisor at Salimetrics LLC (State College, PA, USA) and this relationship is managed by the policies of the conflict of interest committee at the Johns Hopkins University School of Medicine.

## Authors' contributions

ZN participated in data interpretation and manuscript preparation. MG participated in study design and results evaluation. CF performed the statistical analysis. EB participated in study design, graphing, and data work. JS carried out sample collection, treatment, DNA extraction and sample preparation for polymorphism analysis; and participated in results evaluation. DG conceived the study, and participated in project and statistical design, write-up, and coordination. All authors participated in writing and critical review of the manuscript. The final manuscript was approved by all authors.

## Pre-publication history

The pre-publication history for this paper can be accessed here:

http://www.biomedcentral.com/1471-2288/11/170/prepub
